# Improving the uplift capacity performance of the structural skirt foundation by enhancing its properties

**DOI:** 10.12688/f1000research.175992.1

**Published:** 2026-06-23

**Authors:** Aqeel Jaafar Al-zubaidi, A’amal A.H. Al-Saidi

**Affiliations:** 1 University of Baghdad Department of Civil Engineering, Baghdad, Baghdad Governorate, Iraq

**Keywords:** Shallow footing, loose sand, uplift capacity, skirt footing, metal lath, and wings.

## Abstract

**Background:**

Geotechnical engineers are making extensive efforts to improve soil properties and make it suitable and safe for the structures built above it. This research has investigated the use of skirts under shallow footing located on loose sand exposed to uplift loading as a promising method for improving the uplift capacity of soil and a suitable economic alternative to pile foundations.

**Methods:**

A loading device, a square footing model, and two groups of skirts (chamfered and straight corners) with different properties (length, inclination angle, roughness, and wings) of the skirt were used. The performance of un-skirted footing situated on loose sand with 30% relative density was analyzed and compared with skirted footing.

**Results:**

The results showed that adding (Hy-Rib) metal lath and wings to the skirt significantly improved the uplift capacity. For the footing at L/B = 2 (where L is the skirt length and B is the footing width) and a skirt inclination angle of 0°, the maximum values were 7 and 12 times higher than for the un-skirted footing. When the inclination angle was increased to 45° while maintaining the same embedment ratio of L/B = 2, the uplift capacity significantly increased, reaching values of 27 and 20 times for the straight and chamfered corners with the (Hy-Rib) addition, respectively, and 38 and 27 times for the straight and chamfered corners with the wings added.

**Conclusions:**

A significant increase in the uplift capacity of soil was observed by improving its properties by adding a skirt to the shallow footing and making modifications to it using expanded metal lath (Hy-Rib) and wings. The use of skirts has proven to be economically feasible and a successful and promising alternative to the use of uplift piles.

## Introduction

### Soil improvement

In recent years, researchers have been able to find successful alternative foundations for engineering design by improving soil properties (e.g., bearing capacity, settlement rate, resistance to tilt and uplift forces, and reduced permeability) to be in compliance with design standards; they used, for example, geogrid reinforcement, grouting, stone columns, etc. However, it is still governed by the cost and possibility of its implementation on the site. Al-Mosawe et al. ^1^ conducted tests to improve loose sandy soil using a geogrid model. The test results showed an increase in bearing capacity of approximately 22% using one layer of geogrids and 47.5% using two layers.

Jawad ^2^ conducted a study on slope stabilization using stone columns. As a gauge of the possibility of the slope failure, the analysis output is shown as the probability of failure. Because the probability of failure gives a range of values rather than a single value, the study’s results have confirmed that it is a more accurate indicator of slope stability than the factor of safety. Jasim ^3^ investigates the behavior of footing placed on loose sandy soil by conducting a series of experimental tests to improve the bearing capacity of soil by using pomegranate sticks mat as reinforcement. The test results showed that using pomegranate sticks as reinforcement had a clear effect on increasing the soil bearing capacity by four times compared to unreinforced soil.

Sarsam et al. ^4^ conducted a study to test the use of reinforcement technology on gypseous soil in constructing embankments in urban areas with limited space and high costs under the influence of cyclic loads. The tests were conducted on a laboratory model. The results showed that using the soil reinforcement technology led to a 59% improvement in the soil. Shakir ^5^ conducted laboratory tests on gypsum soil using Cutback Asphalt as a method of soil treatment to improve its properties (collapse potential and bearing capacity). Different percentages of Cutback Asphalt used, ranging between (3%–15%), and the results showed that the best value for bearing capacity was used at a percentage of 9%, while the collapse rate was reduced using a percentage of 12%.

Al-Hadidi and AL-Maamori ^6^ conducted experimental work to stabilize gypsum soil using cement as a method of soil treatment, where different proportions of cement were used, ranging between (2%–15%), and the results showed that using a proportion of 10% reduced soil collapsibility by 86%. Salih ^7^ conducted laboratory experiments to improve the properties of clay soil (compaction and strength) using natural stone powder, where different proportions of powder were used. The results showed that cohesion increased by 12%, while the friction angle improved by 21%. Bachay and Al-Saidi ^8^ conducted laboratory tests to explore the optimal number of geogrid reinforcement layers, which was used to improve the soil under ring footings subjected to inclined loads. The results showed that the optimum number of geogrid layers is four, which led to improved tilting of footings.

Mohammed and Al- Saidi ^9^ conducted experimental works to study the behavior of geogrid reinforcement to improve the bearing capacity of loose sandy soil carrying a ring footing. The test results showed that the use of geogrid reinforcement was effective in improving bearing capacity and reducing footing instability under complex loading conditions. Daibil and Al-Saidi ^10^ investigated the behavior of a soil-anchor system under different loading conditions that contribute to improving soil. Researchers found that geogrid reinforcement enhances vertical anchor plate performance, influenced by factors like size, position, water content, and pullout angle.

### Skirted footing

Recently they developed a technique to use skirts, which are made of steel or concrete and fixed at the lower edges of shallow foundations. Skirts have proven to be a successful alternative to deep foundations, as they lead to improved soil properties. Concrete skirted foundations are used for anchoring the subsea production system (SPS). ^11^ In jacket constructions, steel skirting footings have effectively taken the place of pile foundations. ^12^ Suction anchor piles were used in the Nkossa oil field. ^13^

Sherif and Roy ^14^ examined the behavior of suction caissons subjected to static and cyclic pullout loads in sandy conditions. The study examined the influence of the model suction caisson’s length and its performance in drained and undrained scenarios. Zdravkovic et al. ^15^ examined the influence of various parameters on the pull-out capacity of bucket foundations in soft clay. The parameters include load inclination, skirt length, foundation diameter, soil adhesion, and soil anisotropy. The soil is initially considered to be isotropic soft clay, represented by a variant of the modified Cam clay model.

The inclined uplift capability of a suction caisson under both horizontal as well as vertical stress on typically consolidated clay has been investigated by Supachawarote et al. ^16^ The findings demonstrate that the ideal loading depth along the caisson’s centerline is unaffected by the loading direction. The study by Kelly et al. ^17^ found that if the up-wind leg(s) could resist significant tensile loads, the efficiency of multiple footing structures using suction caisson foundations would be improved. In a variety of offshore applications, such as wind turbines, oil platforms, offshore industrial facilities, and jacket structures, skirted foundations have proven to be an efficient substitute for surface, pier, and pile foundations. ^18^

Using the limit analysis theorem, Merifield and Sloan ^19^ conducted a numerical investigation to determine the ultimate pullout force for both vertical and horizontal plate anchors in frictional soils. The investigation showed that the capacity of vertical anchors was significantly impacted by the roughness of the anchor interface. Singh et al. ^20^ investigated the influence of pullout rate on the uplift behavior of plate anchors (70 mm diameter) embedded in soft saturated clay, varying the pullout rate from 1.4 mm/min to 21.0 mm/min. The relationship between breakout force and suction force concerning embedment depth and pull rate is presented. To examine the behavior of suction caissons under a tripod structure, two different kinds of studies were carried out. ^21^ The conventional loading criteria for a tripod foundation were first predicted by improving an already-existing computer program. To investigate how small-scale suction caissons behaved during the loading and installation stages, centrifuge experiments were performed on them.

Zhou et al. ^22^ developed a numerical model to simulate the breakout process of a disk initially resting on the surface of a porous, elastic, saturated seabed. Acosta-Martinez ^23^ offered experimental results from a series of model tests conducted at the beam centrifuge apparatus. This study examined the uplift resistance of shallow skirted foundations for offshore structures exposed to buoyancy or overturning loads. The findings were utilized to examine the mobilization of negative excess pore pressures that improve uplift resistance and their dissipation over time. Kakasoltani et al. ^24^ performed an experimental study on the pull-out performance of tapered suction caissons. Experiments at 1g have been conducted on eight small-scale suction caissons. Ahmadi and Ghazavi ^25^ investigated the uplift performance of skirted foundations with inclined faces was investigated numerically. The data point to a number of broad conclusions. Compared to vertical-faces foundations, the pullout capability of skirted foundations with inclined sides is reached at greater displacements. The bearing capacity rises with the soil’s undrained shear strength (Su), especially for foundations with an inclined skirt surface.

A series of centrifuge model experiments were carried out by Li et al. ^26^ to assess the uplift strength of rectangular-shaped mudmats placed on mildly kaolin clay under overconsolidated conditions. Singh et al. ^27^ claim that adding a structural skirt improves the bearing capacity of a foundation, reduces settlement, and modifies its load-bearing behavior. The bearing capacity of a ring foundation can increase by 11% to 30%. The pullout load on shallow foundations is much higher than their self-weight. Therefore, this study sought to investigate the behavior of skirt foundations with L/D ratios of 0.5, 1.0, 1.5, 2.0, and 2.5 on loose sand under both dry and flooded conditions. The undrained response of skirted shallow foundations to uplift and compression has been modeled through large deformation finite element analysis, as demonstrated by Chatterjee et al. ^28^

A study was conducted by Li et al. ^29^ that revealed the subsequent uplift capacity of shallow skirted foundations built on lightly over-consolidated kaolin clay is influenced by consolidation and compressive preloading.

Shen et al. ^30^ have conducted numerical analyses. The pullout capacity of a skirt foundation is influenced by several elements, including the submerged weight of the skirt, skin friction along the skirt wall, the soil plug within the skirt, the soil tensile strength at the skirt base, and the suction pressure generated from tensile loading across the skirt foundation. ^31^ A recent experimental study conducted in a centrifuge is detailed by Wang et al. ^32^ Xie et al., ^33^ conducted a study examining how the geometry of a skirt influences the uplift capacity of skirt foundations. The geometry, undrained shear strength, and length to diameter ratio significantly affect the uplift capacity of the skirt foundation.

Kulczykowski ^34^ presented findings from 1g model tests conducted under single gravity on a skirted foundation placed in sand and exposed to a rapid uplift load. The impact of displacement rates between 5 mm/s and 450 mm/s on ultimate capacity, suction pressure within the skirt compartment, and extraction duration was examined. Test results demonstrate that the displacement rate significantly influenced the magnitude of uplift resistance and the suction under the foundation lid, while exerting minimal impact on the relationship between stress and foundation displacement.

Given the lack of research related to shallow skirt foundations subjected to uplift forces, conducting research addressing this important and influential aspect is essential. This study investigates the behavior of shallow skirt foundations under uplift loads, resting on loose sand. These skirts increase the soil density by confining it between its inner faces, thus improving its bearing capacity and resistance to uplift forces, which in turn enhances foundation performance. Many experimental works and theoretical studies have been conducted to investigate the performance of shallow skirt foundations subjected to uplift forces.

## Methods

### Research methodology

Figure 1 is a flowchart illustrating the research methodology designed to achieve the objectives of this research. The primary objective of this research is to evaluate the performance of skirted foundations placed on loose sand subjected to uplift loads by making some modifications to its properties to improve uplift capacity and compare them with foundations based on single piles to determine whether it could be a promising alternative to the piles foundations.

**Figure 1.**
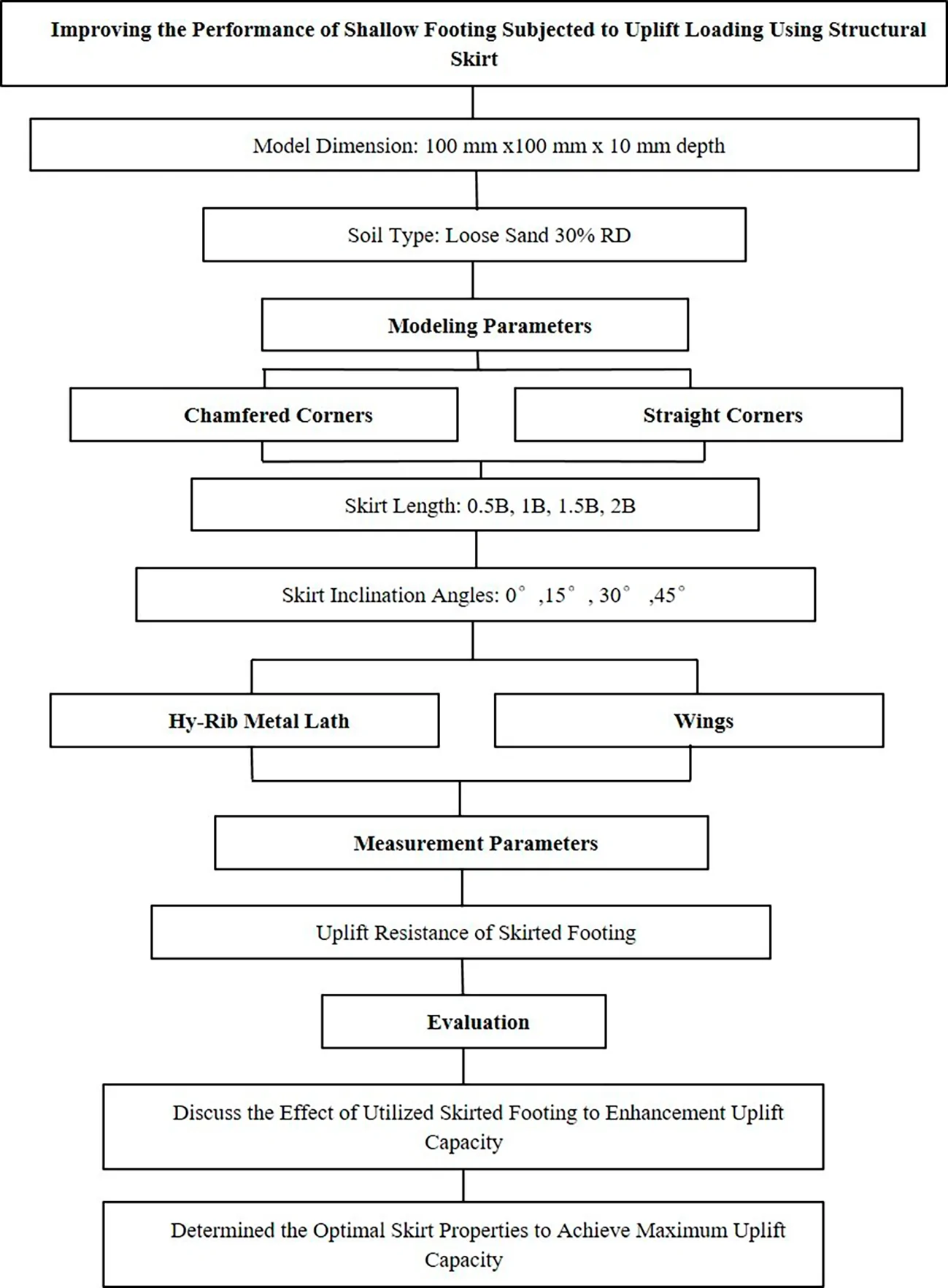
Research Methodology: A flowchart illustrating the research methodology designed to achieve the objectives of this research.

### Model box test

The apparatus used for conducting the tests appears in Figure 2 consisted of a steel box as a container for the soil to be tested, with (60 × 60 × 60) cm. The box was manufactured with steel angles as a frame, while steel plates were used to close the sides and base. A glass panel was used at the front to monitor the behavior of the soil and skirted footing during testing. In addition, the loading system consisted of a steel loading arc manufactured to support the base of the electric hoist (car jack). The hoist was fixed inverted onto the loading arc to meet the testing requirements and connected to a load cell at the other end, equipped with a transformer to regulate the loading speed. The applied loading speed was 0.5 mm/min and was expressed as displacement rate. The load cell was connected to a laptop supplied with a data logger to accurately record the readings obtained during the testing. A sand-raining technique was used, consisting of a plastic cone with a 25 mm opening diameter suspended by a steel rope to facilitate manual lifting of the cone, while lateral movement was performed manually. A ball valve with the same opening diameter as the cone was located at the end of the cone to regulate the sand flow rate, which helped achieve the necessary height to obtain the desired relative density. The footing uplift displacement was measured using two linear variable differential transformers (LVDTs). The transformers were connected to a data logger.

**Figure 2.**
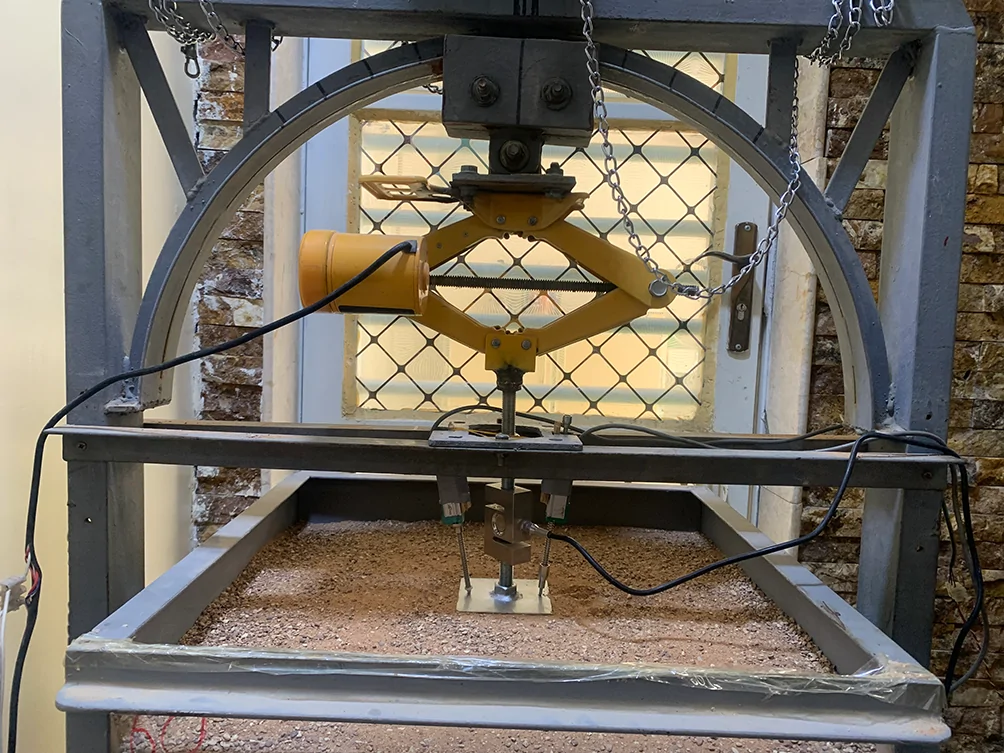
Testing apparatus: The apparatus used for conducting the tests.

### Model soil test

This experimental study was conducted utilizing the sands of Karbala. The sand was cleaned, dried, and subsequently sieved through a 4.75 mm sieve before the test. Figure 3. displays the particle size distribution of the used sand. The particle size distribution of the sand utilized was analyzed in accordance with ASTM (D422-63) standard, as depicted in Table 1.

**Figure 3.**
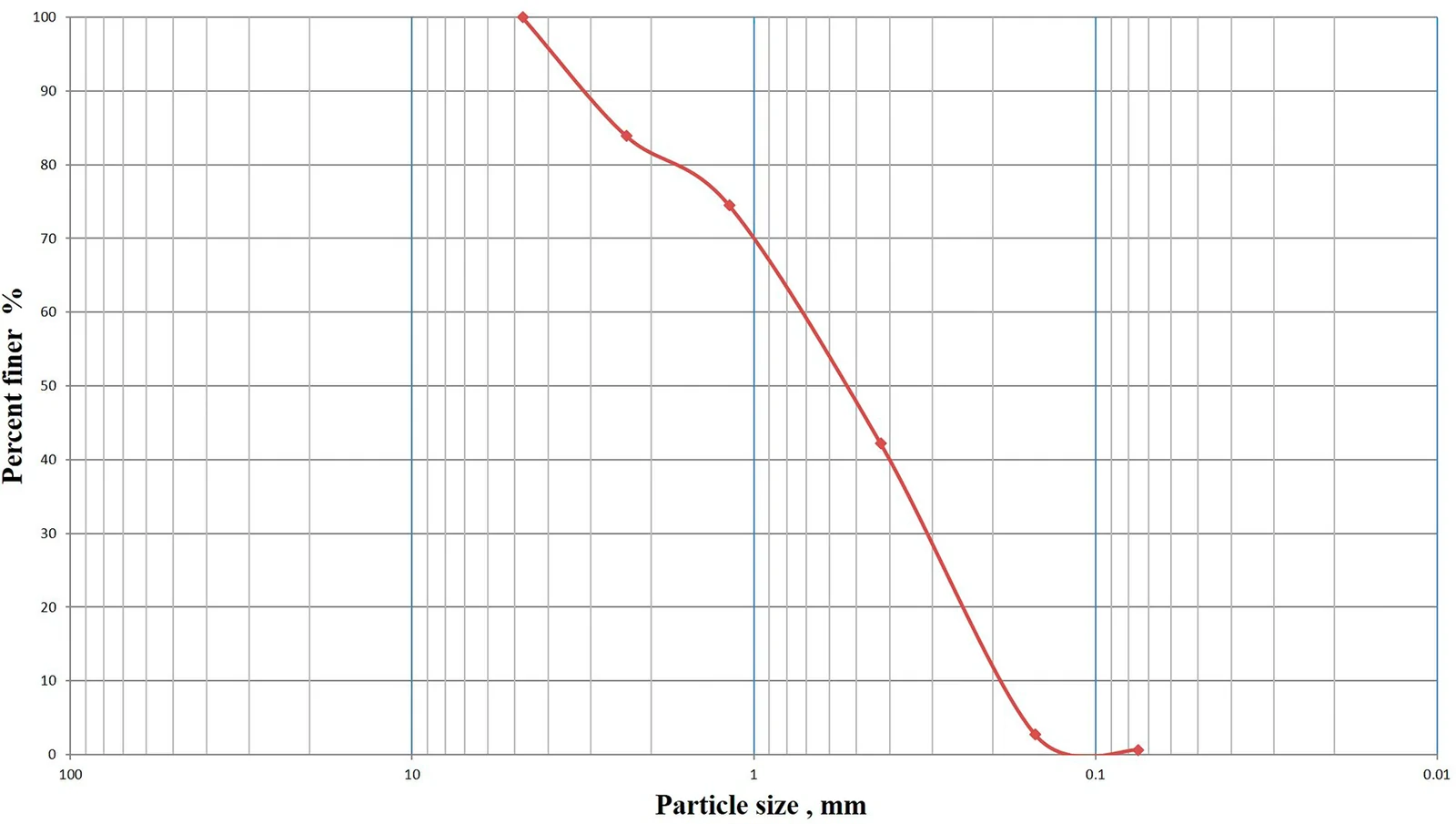
Grain size distribution of the sand: Displays the particle size distribution of the used sand.

**Table 1.** Physical properties of used sand.

Property index	Value	Specifications
Specific gravity (Gs)	2.651	ASTM D-854-92 (ASTM D854–14. 2014)
D10 (mm)	0.2	
D30 (mm)	0.34	
D60 (mm)	0.84	ASTM D-422
Coefficient of uniformity (Cu)	4.188	
Coefficient of curvature (Cc)	0.684	
Maximum dry unit weight (KN/m ^3^)	18.56	
Minimum dry unit weight (KN/m ^3^)	16.09	(ASTM D 4253) & (ASTM D 4254)
Dry unit weight in test (KN/m ^3^) at R.D = 30%	16.76	
Relative density (R.D)%	30	(ASTM D 2049–64)
The angle of interior friction Ø at R.D = 30%	33.7°	(ASTM D3080)
Soil classification (USCS)	Poorly graded sand (SP)	Unified soil classification system

To achieve the required relative density for the tests, the raining technique was used. To reach the specified level, the container was filled with sand in five layers, each 100 mm thick. This system was developed to achieve this goal. This technique consists of a plastic cone with a 25 mm opening diameter, connected to three steel ropes for balance. This rope acts as a manual lever to facilitate the upward movement of the cone, while the lateral movement was manually executed to distribute the sand evenly across all corners of the container. A ball valve, fixed at the cone’s exit, to regulate the sand flow rate. Sand flow rate and raining height affect the sand density in the raining technique. ^35^ The relative density was maintained at 30% in all experiments to investigate this effect on loose sand. The required level to achieve the required 30% density is 224 mm.

### Model skirted footings

A square footing of (10 cm * 10 cm) dimensions and a thickness of 1cm has a nut with a size of 2.7 cm fixed into the surface of the footing model by a welding technique that is used to connect the footing with the loading arm. Four disks and screws are welded on each side of the skirt to connect it with the footing by corresponding disks that are welded on each side of the footing by nuts, and tiny holes are drilled into the surface of the footing to prevent the (LVDT) from shifting position, as shown in Figure 4. A steel skirt of 10 cm × 10 cm in dimensions and 2 mm thick was used; the depth of embedment was (0.5, 1, 1.5, and 2) B (where B = width of footing). Skirt inclination angles (0°, 15°, 30°, and 45°) with two types of corners: chamfer & straight corners. Some modifications were made to both types by increasing the roughness of the inner surface of the skirt using Hy-Rib metal lath material. Figure 5 and Table 2 showed the physical properties of it, and wings were added to the lower edges of the skirt as illustrated in the Figures 6 & 7.

**Figure 4.**
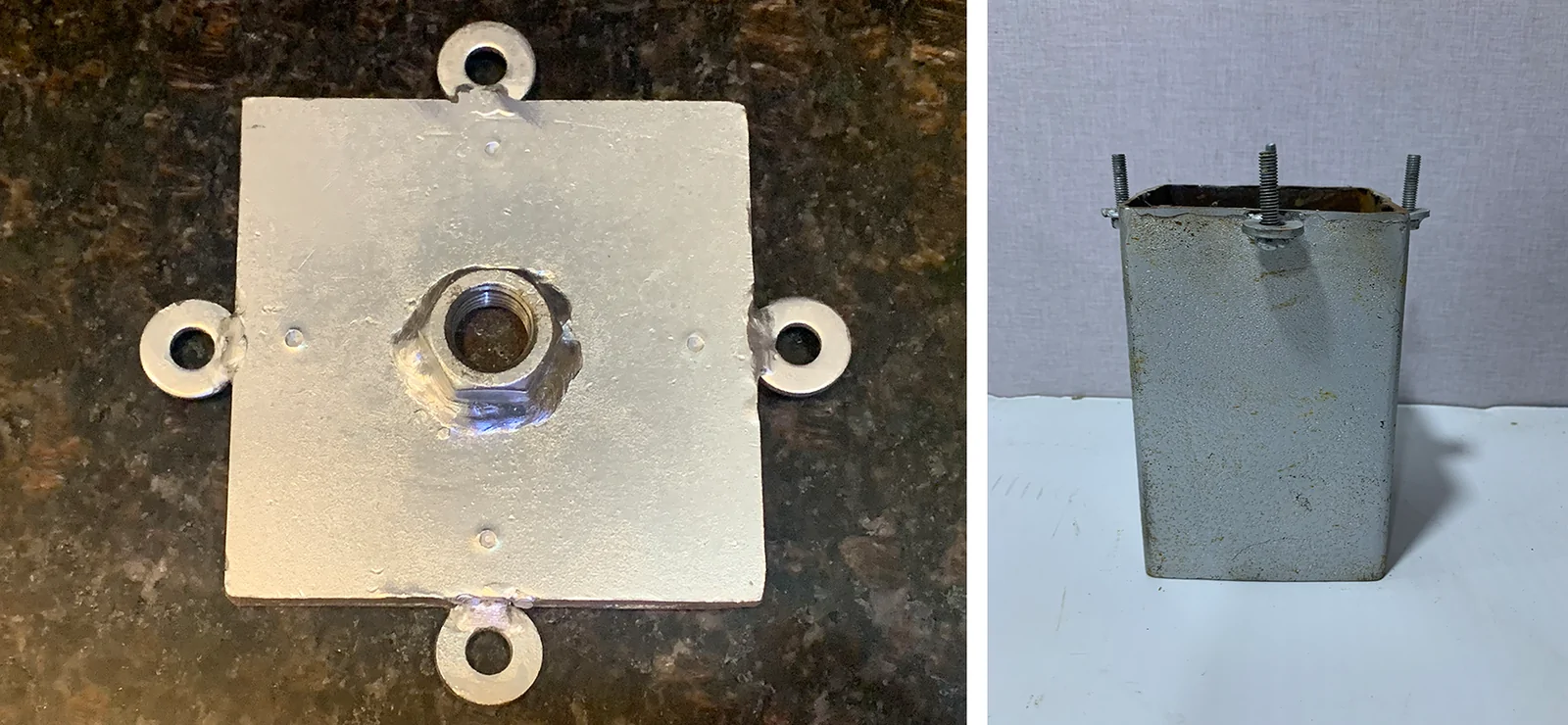
Model Skirt & Footing: A square footing of (10 cm*10 cm) dimensions and a thickness of 1 cm has a nut with a size of 2.7 cm fixed into the surface of the footing model.

**Figure 5.**
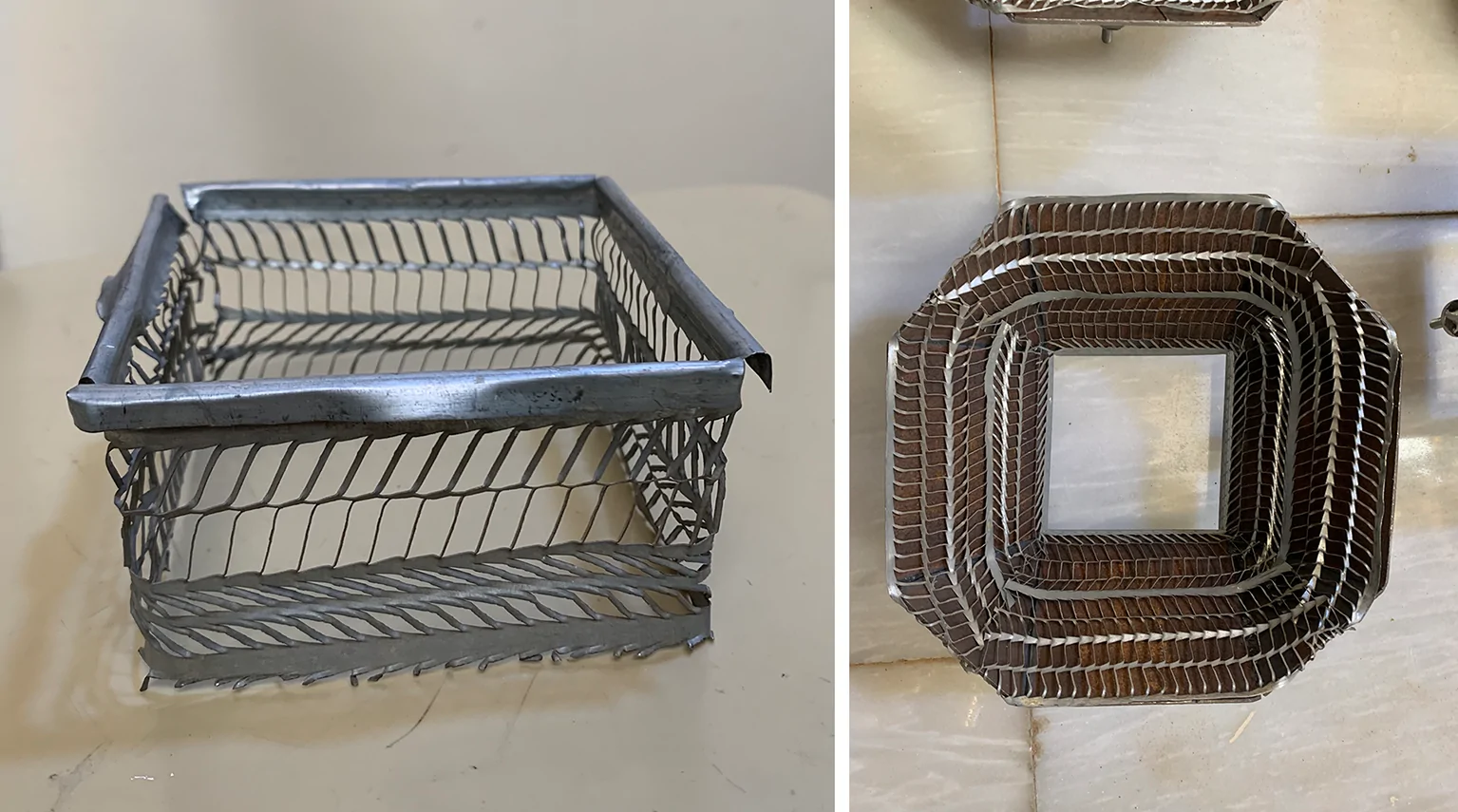
Hy-Rib metal lath: Increasing the roughness of the inner surface of the skirt using Hy-Rib metal lath material.

**Table 2.** Physical properties of Metal Lath (Hy-Rib).

Item	Details
Model	Rib Lath
Material	Cold rolled and hot galvanized steel in accordance with ASTM C 847.
Rib Type	7 Rib
Rib Distance	100 mm
Rib Height	8 mm
Rib Lath Thickness	0.2 mm
Sheet size	600 mm × 2400 mm
Hole Size	6 mm × 10 mm
Weight	1.79 (kg)

**Figure 6.**
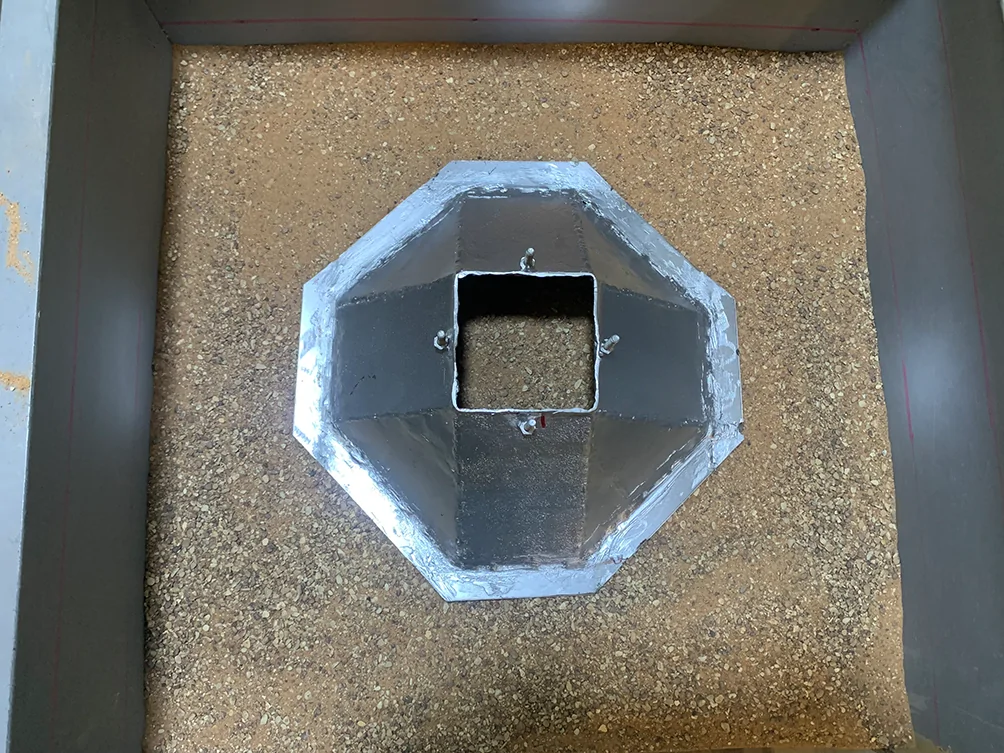
Chamfered corners skirted model with wings: wings were added to the lower edges of the skirt.

**Figure 7.**
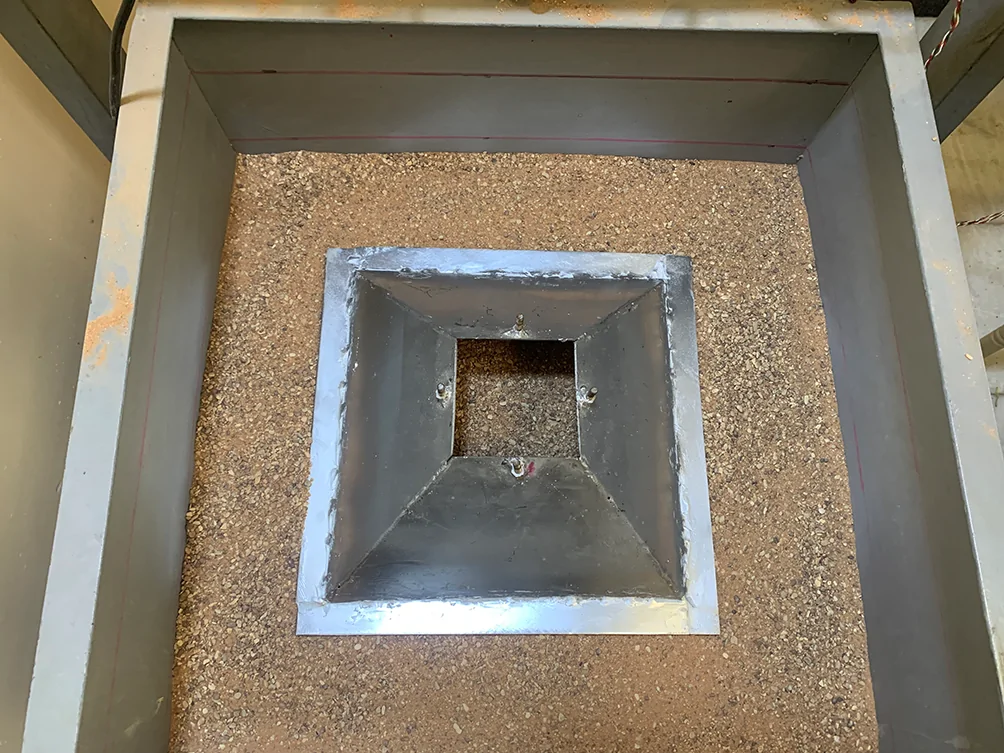
Straight corners skirted model with wings: wings were added to the lower edges of the skirt.

### Failure criteria

The criterion for failure uplift capacity employed in all model tests is the local shear failure, as defined by Terzaghi. ^36^ The foundation’s maximum uplift capacity per unit length will be achieved when an angle α is established by the failure surface in the soil in relation to the horizontal at ultimate load. The relative density and the soil’s friction angle influence the value of α, which is 90° − 1/2 ϕ. ^37^

## Results and Discussions

Besides the impact of the skirt geometry, embedment ratio (L/B), and skirt inclination angles on the pullout capacity, the primary parameters examined in this research are the impact of roughness of the inner sides and adding wings at the lower edges of the skirt to increase anchoring in the soil. A total of (49) laboratory experiments were carried out, comprising one test for the unmodified footing, 24 tests for the footing with chamfered corners, and 24 tests for the footing with straight angles (12 tests with wings and 12 tests without) for both types above. Regarding the embedment ratio (L/B), all experiments considered the ratios (0.5, 1, 1.5, and 2) as well as the skirt inclination angles (0°, 15°, 30°, and 45°).

### The Effects of Hy-Rib lath on model skirt

The uplift load of the unskirted footing was used for comparison with that generated by the footing that was improved by adding a skirt. Where the uplift force was 13.88 N. Preliminary tests examined the effect of increasing the roughness of the skirt by adding metal lath (Hy-Rib) to the inner sides of the skirt at different embedding ratios (L/B) and different inclination angles on the uplift capacity. The results showed that the uplift capacity of shallow skirted footing generally increased with increasing embedding ratio (L/B) and inclination angles.

Figures 8– 10 show the load-displacement relationships for shallow skirted footing on loose sand. The maximum uplift load was observed at 26.51 N at L/B = 0.5, which is approximately 1.9 times greater than that of the unskirted footing and 7.2 times for L/B = 2, with a peak load resistance of 99.74 N at a skirt inclination angle of 0°. Hy-Rib clearly demonstrates the effect of shape, increased embedding ratio, and skirt inclination angles on the uplift load. The uplift load for the skirted footing was observed at L/B = 0.5 and an inclination angle of 15° was 30.98 N and 29.86 N which is equal to 2.23 and 2.15 times for the chamfered and the straight-corner, respectively, compared to the unskirted footing. For the same embedding ratio, the uplift load increased by 35 N for both skirt types when the skirt inclination angle was raised to 45°, which is 2.5 times greater than that of the unskirted footing. The greatest increase in uplift resistance was observed at L/B = 2 and an inclination angle of 45°.

**Figure 8.**
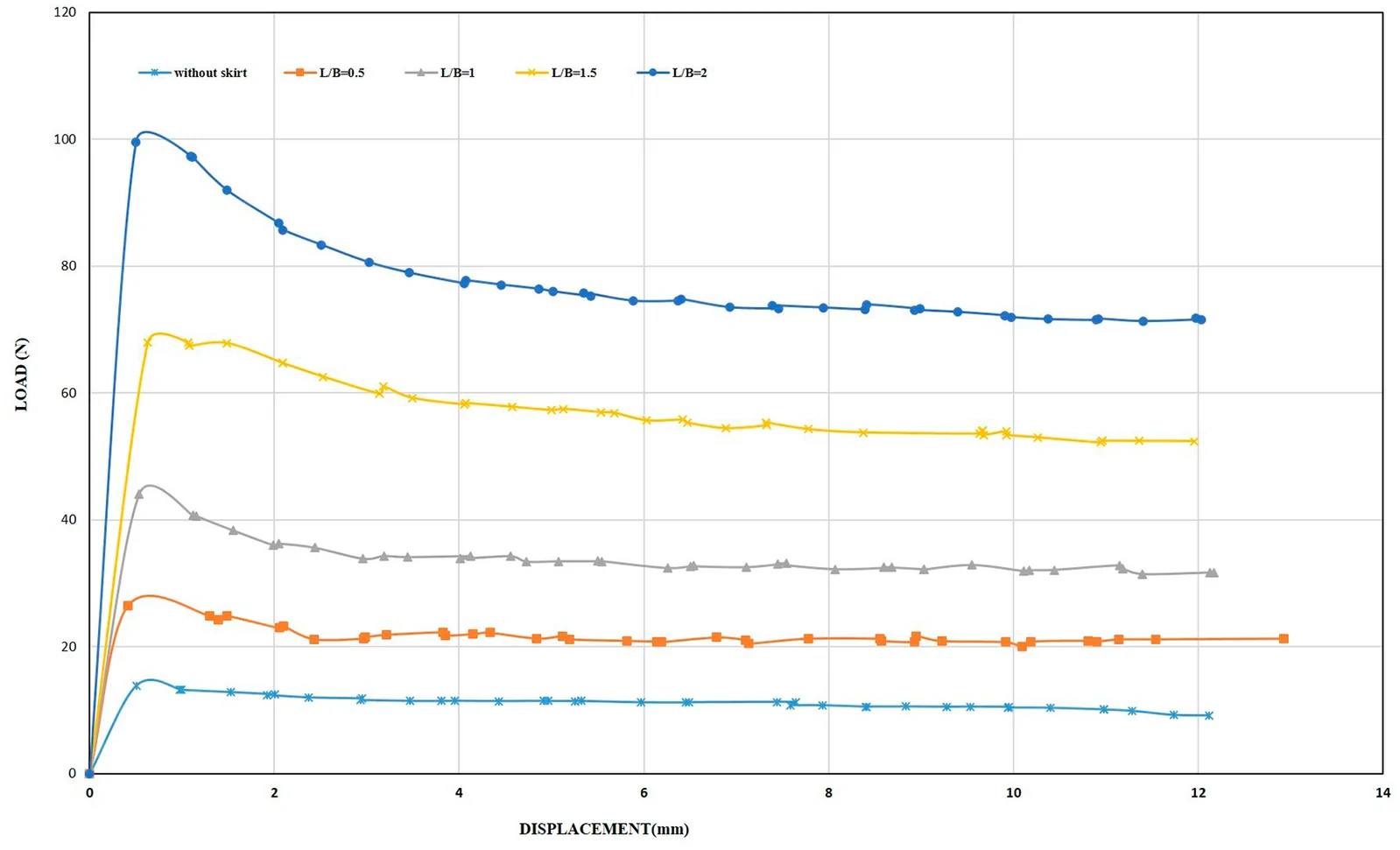
Skirted footing with Hy-Rib metal lath: the load-displacement relationships for shallow skirted footing on loose sand.

**Figure 9.**
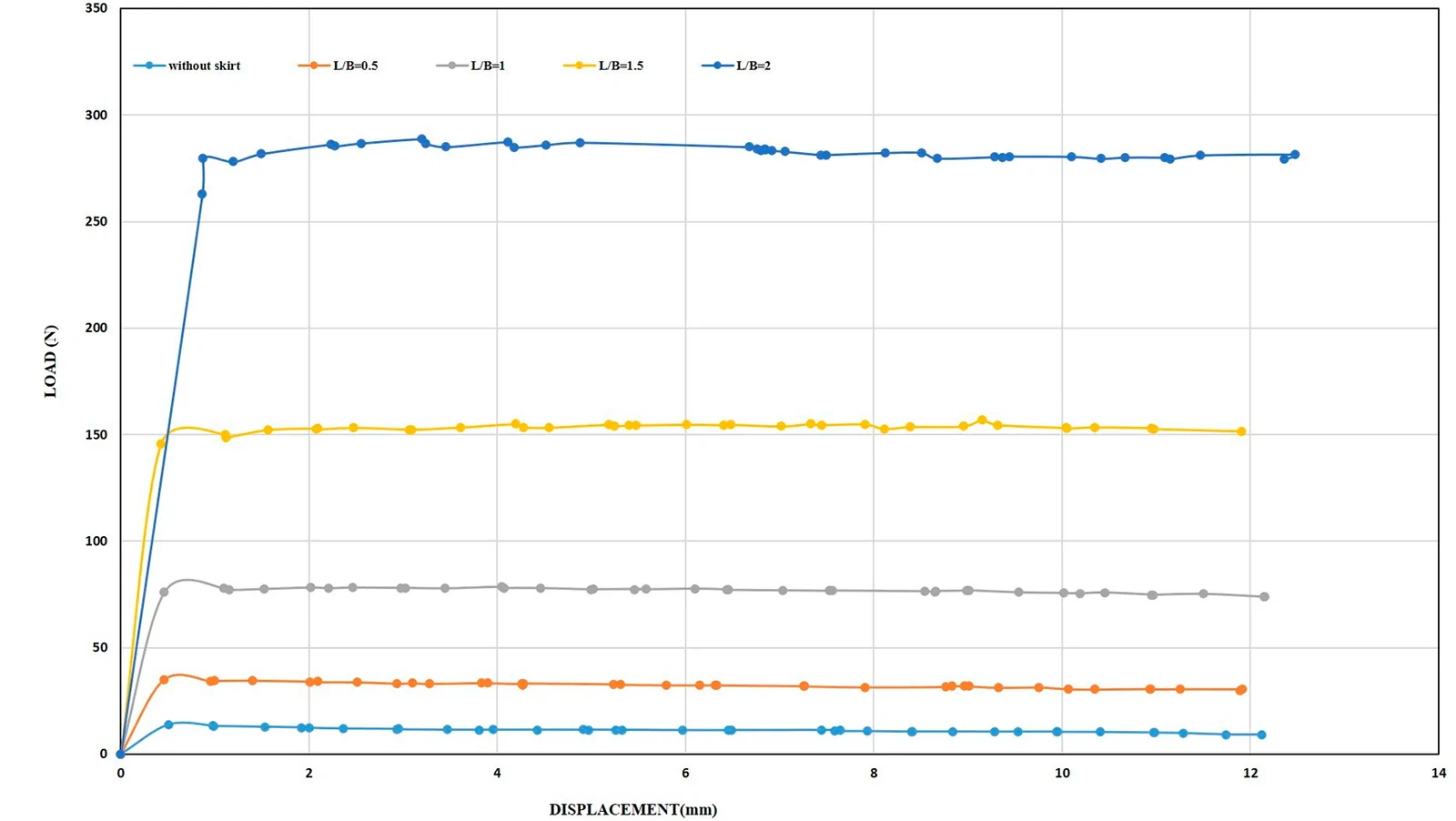
Chamfered-corner skirted footing with Hy-Rib metal lath and α = 45°: the load-displacement relationships for shallow skirted footing on loose sand.

**Figure 10.**
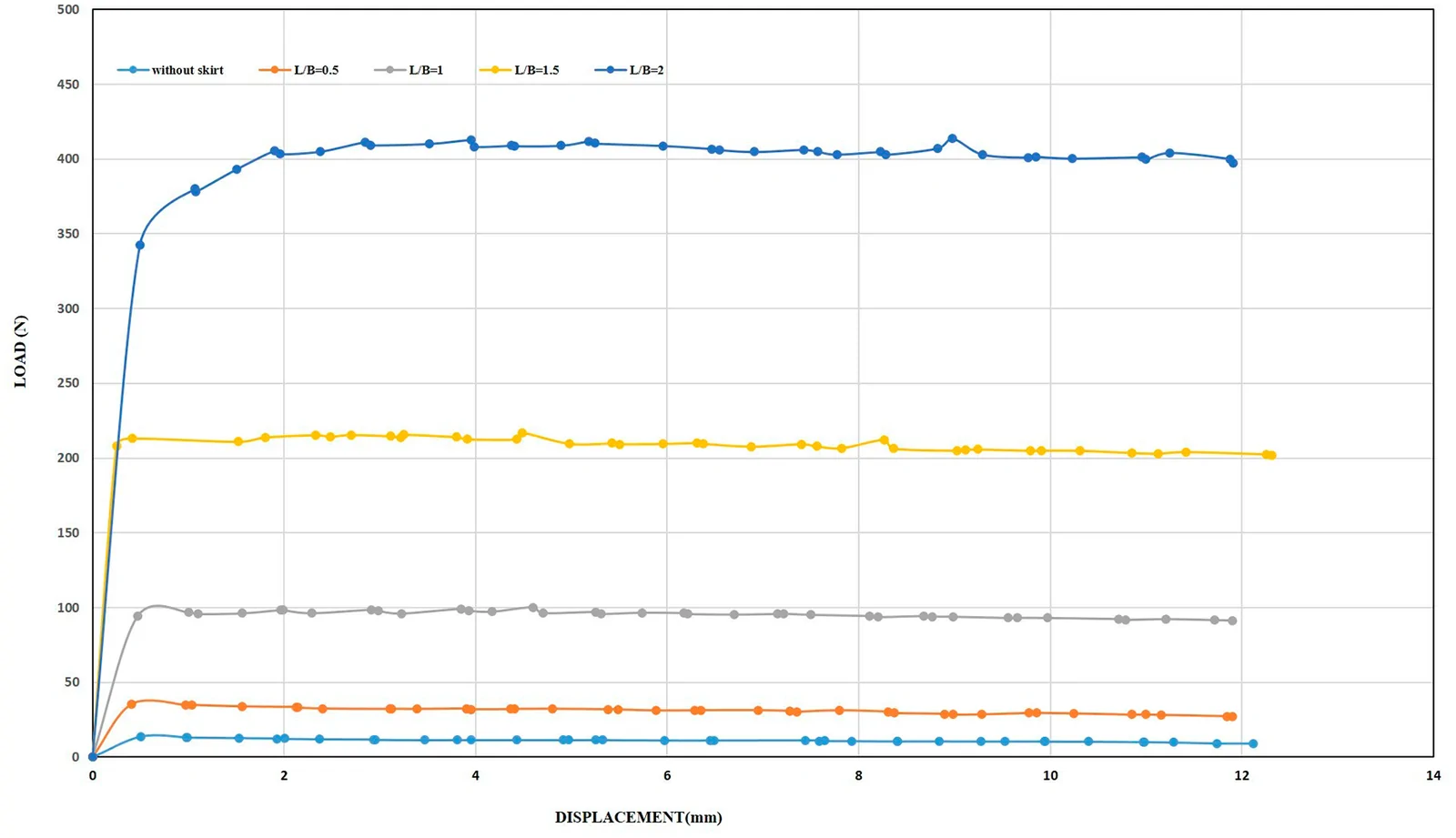
Straight-corner skirted footing with Hy-Rib metal lath and α = 45°: the load-displacement relationships for shallow skirted footing on loose sand.

Increasing the embedding ratio is a fundamental concept in skirt design because it increases the friction surface area between the soil and the skirt, while increasing the skirt inclination angle increases the skirt’s stability in the soil by acting as an anchor. In addition, results showed that the uplift was 176 N at a 15° inclination angle and L/B = 2 for the chamfered-corner and 197 N for the straight-corner, which are 12.7 times and 14.2 times, respectively, compared to the unskirted foundation. The uplift load increased to 279.8 N and 380 N when the inclination angle increased to 45° and L/B = 2 which is 20 times greater than the unskirted footing.

The variation in results can be observed due to using the Hy-Rib, which is supposed to be linear. However, this variation is due to the granular composition of the sandy soil used. The maximum particle size must be smaller than the openings of the Hy-Rib layer in order to achieve fully confine the soil. Therefore, the presence of particles larger than the openings prevents smaller particles from entering through the openings, thus creating a gap between the Hy-Rib layer and the skirt wall. Since the granular composition of the soil cannot be controlled, laboratory tests such as sieve analysis are used to determine the maximum particle size, and accordingly, Hy-Rib layers are prepared to be larger than the largest sand grain.

The uplift load of the skirt with 0° inclination angles showed a different behavior than that of the skirt with different inclination angles for both the chamfered and straight skirt types, as the uplift load reaches its peak value with a very diminished displacement and then begins to decline linearly. However, the skirt with different inclination angles, as the maximum uplift load is achieved at a very diminished displacement as well, then decreases; after that, the uplift resistance begins to recover gradually, reaching values higher than the uplift value at the beginning of the test. This can be explained by the fact that increasing the inclination angle and the skirt length, under the influence of the uplift load and the weight of the soil itself, will lead to a rearrangement of the soil particles and a densification of the soil, which is similar to the concept of consolidation in terms of compression condition. Therefore, this phenomenon can be used as a safety factor in resisting the foundation to total failure after the initial failure, which is the local shear failure, and then can avoid sudden whole failure of the foundation.

The maximum load consists of the weight of the standard footing, the weight of the soil plug inside it, and the friction between the sand and the outer surface of the footing. Table 3. shows the percentage improvement of the soil for skirted footing enhanced with (Hy-Rib) compared to the unskirted footing.

**Table 3.** Uplift capacity values of skirted footings with (Hy-Rib) metal lath.

Skirt without modification B (mm)	Bearing capacity (N)	Skirt length L (cm)	Skirt inclination angle (α)	Bearing capacity (N)	Improvement %
100	13.88	0.5B	0°	26.52	191.00
		1B		44.09	317.58
		1.5B		67.98	489.66
		2B		99.59	717.32

The principle of the skirt is to confine the soil between its walls. This is achieved through the friction of the soil against the sides of the skirt. The results obtained are consistent with those of most researchers specializing in the field of skirting. Therefore, greater roughness results in increased frictional force, which in turn increases the soil’s ability to withstand pressure and its uplift force in tension. Note that frictional force is the primary force used in uplift capacity.

These results comply with the results obtained by Al-Aghbari and Mohamedzein ^38^ that a variety of parameter combinations, including foundation depth, skirt side friction, skirt thickness, and skirt depth. In order to focus on the behavior of skirted foundations placed on clay under both uplift and compression stresses, Acosta-Martinez et al. ^39^ conducted centrifuge experiments. According to this study, employing a skirted foundation improves resistance to compression and uplift stresses.

Gaaver ^40^ also found that the skirt wall side provides resistance to the skirted footing subjected to uplift loading. Tripathy ^41^ demonstrated that in order to reduce settlement and increase bearing capacity, a rough-skirted foundation is preferable to a smooth-skirted one. Increased bearing capacity and less settling of shallow foundations are strongly correlated with skirt depth and roughness, whereas relative density has a variety of effects. ^42^

Gnananandarao et al. ^43^ showed that adding a skirt to the multi-edge T-shaped footing significantly improved the bearing capacity ratio in both partial and total roughness scenarios.

Shaft resistance is a significant source of pile capacity under axial force in cohesionless soils, particularly when the pile is subjected to uplift loading.

Skirt angle and surface roughness decrease settling and raise the bearing capacity. ^44^

Tables 4 and 5 showed all results of uplift load obtained for skirted footing modified by adding Hy-Rib metal lath.

**Table 4.** Uplift capacity values of Chamfer-corner skirted footings with (Hy-Rib) metal lath.

Skirt without modification B (mm)	Bearing capacity (N)	Skirt length L (cm)	Skirt inclination angle (α)	Bearing capacity (N)	Improvement %
100	13.88	0.5B	15°	30.98	223.16
			30°	31.42	226.34
			45°	35.05	252.41
		1B	15°	62.73	451.82
			30°	70.46	507.51
			45°	77.96	561.49
		1.5B	15°	116.82	841.38
			30°	145.46	1047.67
			45°	150.04	1080.69
		2B	15°	176.51	1271.32
			30°	237.00	1707.00
			45°	279.80	2015.27

**Table 5.** Uplift capacity values of Straight-corner skirted footings with (Hy-Rib) metal lath.

Skirt without modification B (mm)	Bearing capacity (N)	Skirt length L (cm)	Skirt inclination angle (α)	Bearing capacity (N)	Improvement %
100	13.88	0.5B	15°	29.86	215.08
			30°	36.44	262.43
			45°	35.44	255.24
		1B	15°	63.66	458.51
			30°	81.40	586.30
			45°	96.77	696.96
		1.5B	15°	147.13	1059.74
			30°	184.56	1329.28
			45°	213.07	1534.65
		2B	15°	197.52	1422.62
			30°	317.36	2285.76
			45°	380.02	2737.13

### The effects of adding wings on skirted footing

The parameters that were used for the skirt were modified by adding (Hy-Rib). It is used with a skirt modified by adding wings for both types of skirts(chamfered and straight corner) with the same embedment ratios and inclination angles. A 0.25 B dimension of wings was added to the lower edges of the skirt; such a modification gave high values for the uplift resistance compared to using the Hy-Rib metal lath. Regarding the skirt’s behavior under the influence of the uplift force. It is similar to its behavior in the case of using the Hy-Rib metal lath for both types of skirt by increasing L/B and inclination angles.

Figures 11– 13 show the load-displacement relationships for shallow skirted footing modified by adding wings.

**Figure 11.**
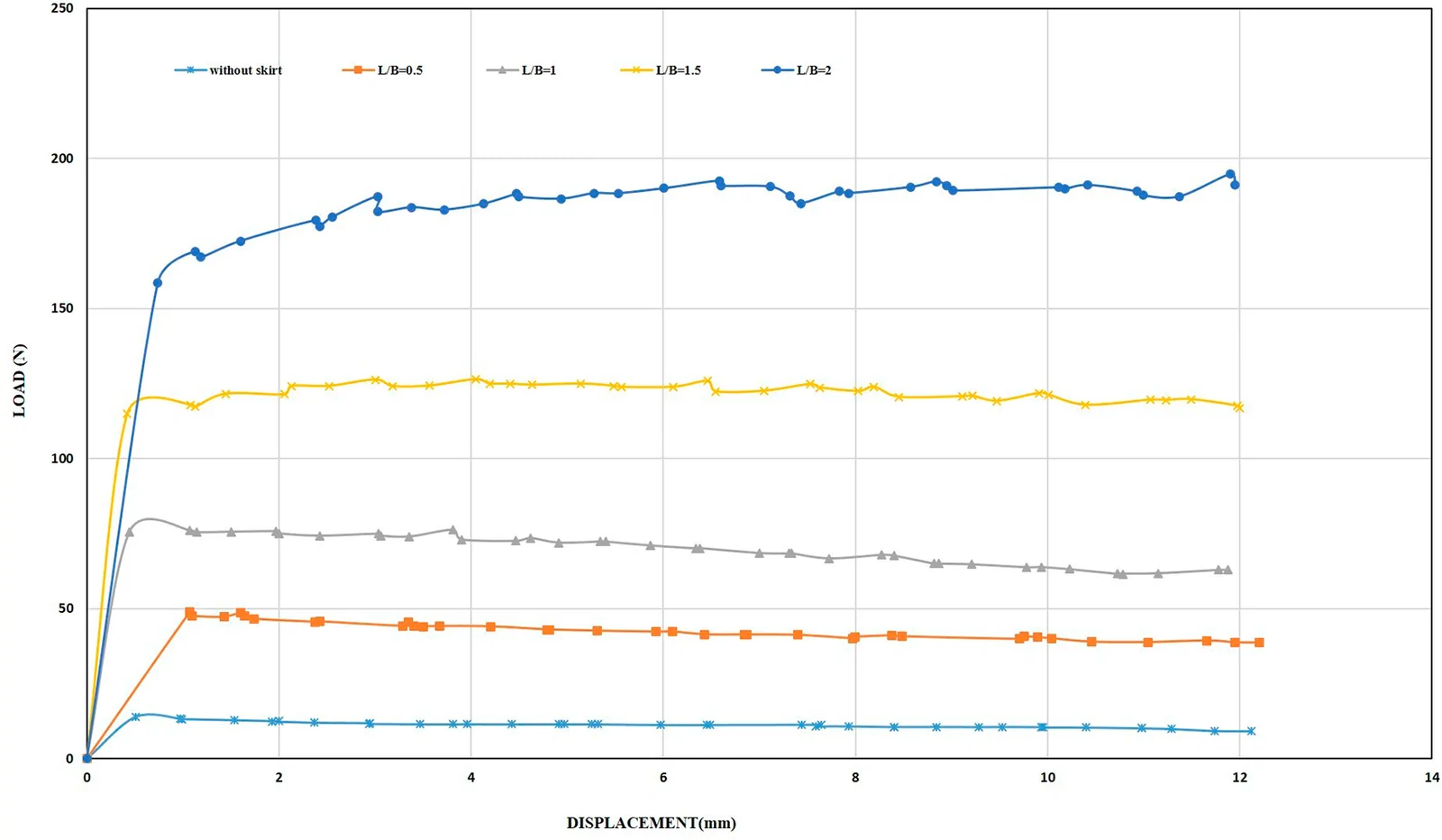
Skirted footing with wings: the load-displacement relationships for shallow skirted footing modified by adding wings.

**Figure 12.**
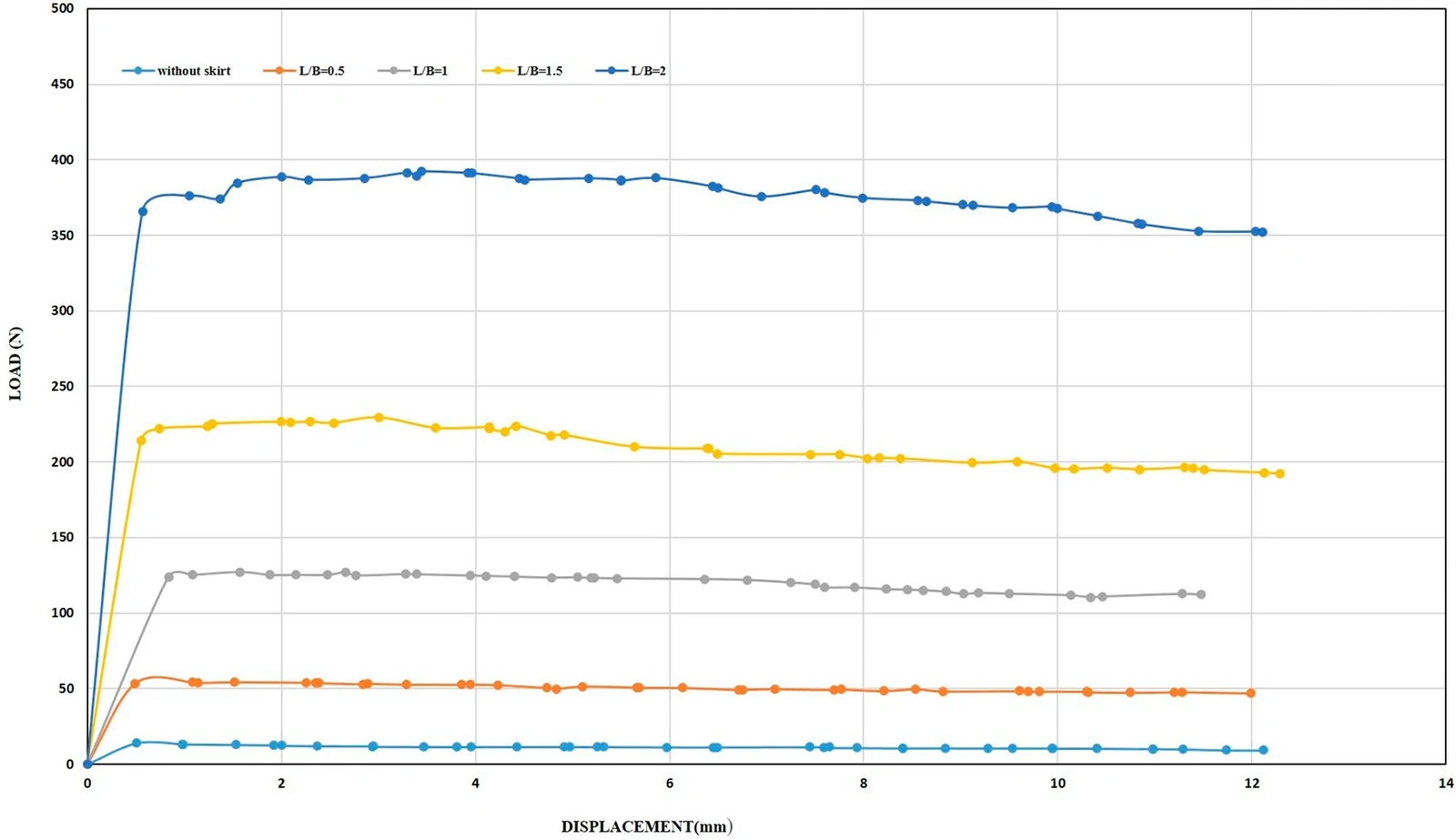
Chamfer-corner skirted footings with wings and α = 45°: the load-displacement relationships for shallow skirted footing modified by adding wings.

**Figure 13.**
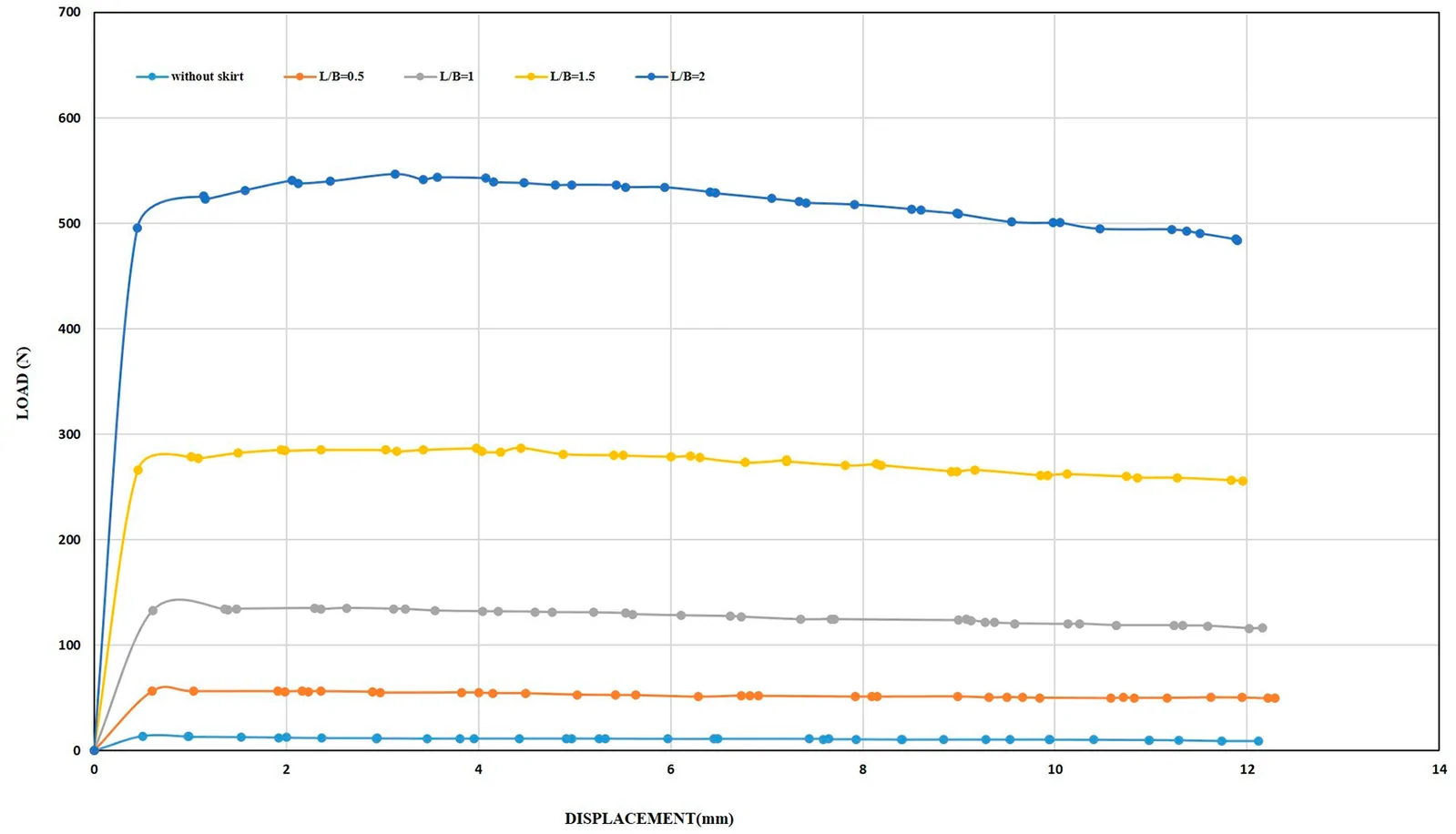
Straight-corner skirted footing with wings and α = 45°: the load-displacement relationships for shallow skirted footing modified by adding wings.

Results show uplift resistance recorded at 48.79 N for the skirt with an inclination angle of 0° and L/B=0.5, which gives improvement ratios 3.51 times the footing without a skirt and 1.84 times the skirted footing modified by Hy-Rib. The uplift resistance continued to rise until it reached 169.07 N L/B=2 and remained at an inclination angle of 0°, which gave improvement ratios of 12.18 times the footing without a skirt and 1.69 times the skirted footing modified by Hy-Rib. The effect of increasing the skirt inclination angle on the uplift resistance of the skirt with wings is clearly evident compared to the Hy-Rib skirted footing. The uplift force for the chamfered and straight skirts at 15° inclination angles and an embedding ratio of 2 was 251.80 and 303.82, which are 18 and 22 times greater than the footing without a skirt. With increasing inclination angles up to 45°, the results indicated 376 N for the chamfered skirt and 525.59 N for the straight skirt, which are 27 and 37.86 times, respectively, greater than the unskirted footing.

The reason for the increased uplift resistance is that the additional wings will anchor the skirt to the ground, and the inclination of the skirt at various angles also serves as an anchor and increases the surface area of the skirt. Also, the increased embedment ratio increases the frictional force between the soil confined within the skirt and the surrounding soil. Results obtained from the added wings to the skirt comply with some researchers regarding cost and working mechanism as follows.

The pullout resistance of belled piles is composed of two parts:

The bell portion’s pullout resistance, which is created by the combination of the earth’s stress on top of the belled section and the skin friction of the surrounding soils, and the drilled shaft’s pullout resistance, which is caused by frictional forces along the pile’s straight section. The ultimate uplift capacity of an under-reamed pile is significantly greater than that of a conventional straight pile. ^45^

The soil between the bells behaves as a pile part, increasing the pile’s effective diameter. ^46^ A belled pile with a suitable length and diameter is better economically than a friction pile (increase in diameter and size). Belled and multi-belled piles can increase the pile shaft’s end (bottom) resistance and decrease negative skin friction. ^46^

In the belled piles, the soil above the bell interacts with the pile. Skin friction rises linearly to a constant value, according to Mohamed and Amr, ^47^ dependent on the L/D ratio of the pile and the sand’s density. Because of the interaction between the pile and the earth, belled pile uplift behavior is more challenging. ^48^

The bells provide sufficient anchorage according to the fundamental principle of the belled pile technique. ^49^

The operation and failure mechanism of the skirt and the straight or bell-shaped uplifting piles are similar, as can be seen from the above. The skirt is a technique to improve the soil, whereas the piles are deep foundations because the failure surface in shallow and deep foundations is the same.

Tables 6, 7, and 8 showed all results of uplift load obtained for skirted footing modified by adding wings.

**Table 6.** Uplift capacity values of skirted footings modified by adding wings.

Skirt without modification B (mm)	Bearing capacity (N)	Skirt length L (cm)	Skirt inclination angle (α)	Bearing capacity (N)	Improvement %
100	13.88	0.5B	0°	48.79	351.39
		1B		76.13	548.31
		1.5B		117.92	849.35
		2B		169.07	1217.72

**Table 7.** Uplift capacity values of Chamfer-corner skirted footings with wings.

Skirt without modification B (mm)	Bearing capacity (N)	Skirt length L (cm)	Skirt inclination angle (α)	Bearing capacity (N)	Improvement %
100	13.88	0.5B	15°	51.07	367.83
			30°	52.67	379.37
			45°	54.26	390.82
		1B	15°	93.81	675.71
			30°	120.94	871.11
			45°	127.14	915.79
		1.5B	15°	171.31	1233.92
			30°	216.22	1557.38
			45°	226.54	1631.67
		2B	15°	251.79	1813.57
			30°	356.62	2568.57
			45°	376.00	2708.16

**Table 8.** Uplift capacity values of Straight-corner skirted footings with wings.

Skirt without modification B (mm)	Bearing capacity (N)	Skirt length L (cm)	Skirt inclination angle (α)	Bearing capacity (N)	Improvement %
100	13.88	0.5B	15°	50.16	361.34
			30°	54.94	395.73
			45°	56.18	404.66
		1B	15°	101.14	728.47
			30°	125.43	903.45
			45°	134.07	965.65
		1.5B	15°	179.22	1290.87
			30°	258.35	1860.81
			45°	278.60	2006.67
		2B	15°	303.81	2188.23
			30°	433.31	3120.94
			45°	525.59	3785.58

**Symbols**
L: skirt length
B: footing width
α: alpha the angle of inclination
D: diameter of skirted
RD: relative density
Ø: angle of internal friction

## Conclusions

1. Increasing the embedment ratio increases the uplift capacity of shallow-skirt foundations by 7 and 12 times for skirted footing with(Hy-Rib) and wings respectively.
2. The uplift capacity of shallow-skirted foundations increases with increasing skirt inclination angles by 20 and 27 times for chamfered skirted footing with (Hy-Rib) and wings respectively and for straight skirted footing was 27 and 38 times with (Hy-Rib) and wings respectively.
3. Increasing skirt inclination angles has a greater effect on soil uplift capacity than increasing embedment ratio.
4. The skirt’s angle of inclination acts as an anchor to secure the skirt to the soil.
5. Increasing the skirt’s roughness by adding a (Hy-Rib) lath material led to an increase in uplift capacity.
6. The skirt’s shape had a significant effect on uplift capacity, with values being higher for straight-corner skirts than for chamfered-corner skirts.
7. The addition of wings to the skirt resulted in significant improvements in uplift capacity, acting as an anchor to secure the skirt to the soil.
8. The load-displacement behavior of the skirt foundation on loose sand is nearly similar in all conditions in general trend.
9. The performance of skirted footings with inclination angles shows behavior that is largely consistent with that of belled piles; therefore, skirt footings can be considered a promising alternative to belled piles.
10. The best location of a bell is at the base of the pile, but for the skirted footing, the skirt connects to the footing at a shallow depth and extends to the specified depth.

## Data Availability

Zenodo: Laboratory Test Results for Sandy Soil Properties. https://doi.org/10.5281/zenodo.18205878. ^50^ This project contains the following underlying data:
• Data Availability Statement.xlsx (Dataset containing results for Direct Shear Test, Sieve Analysis, Relative Density, Specific Gravity, and Raining Technique). Data are available under the terms of the Creative Commons Attribution 4.0 International license (CC-BY 4.0).
