# Supplementary material for: Improving the Uplift Capacity Performance of the Structural Skirt Foundation by Enhancing its Properties
Source: F1000Res. 2026 Aug 24;15:995. Originally published 2026 Jun 23. [Version 2] doi: 10.12688/f1000research.175992.2 (PMC13680842; doi:10.12688/f1000research.175992.2)
Supplement: Peer review history

# Reviewer report: Reviewer response for version 2

**DOI:** 10.5256/f1000research.208648.r524534

**Published:** 2026-10-19

**Authors:** Shaymaa Tareq Kadhim

**Affiliations:** 1 University of Technology-Iraq,, Baghdad, Iraq

This study investigates experimentally the uplift capacity of skirted shallow foundations placed on loose sand, considering different factors such as skirt length, roughness, inclination angle, and wings. The following comments should be addressed to strengthen the paper:

1. Please note that “capability” was used instead of “capacity” on page 4. In order to maintain consistency, unify the terminology “pullout capacity” throughout this paper.
2. On page 4, L and D are not defined upon their first mention in paper. In addition, the footing width (B) in the flowchart needs to be defined.
3.  On Page 5, there is a logical contradiction in the last section of introduction part between the opening sentence “Given the lack of research related to shallow skirt foundations subjected to uplift forces,…” and the closing sentence “Many experimental works and theoretical studies have been conducted to..” please clarify this paragraph.
4. The objective statement needs to be modified to focus on research contribution.
5. The study uses small scale laboratory model tests. Scale effect and boundary influence need to be discussed in this paper.  Discuss whether the testing box boundaries affect the failure surface and the effect of sand grain size relative to the model size.  
6. In Figures 6 and 7, it is recommended to add detailed dimensions, such as footing width, skirt depth, wing width, etc. Providing schematics with full details of dimensions for both types of skirt footings is highly recommended to enhance clarity.
7. In Figures 8 and 11, the angle α should be explicitly defined.
8. Incomplete/grammatically ambiguous sentence on page 11:  “The uplift load increased to 279.8N and 380 N when the inclination angle increased to 45° and L/B= 2 which is 20 times greater than the unskirted footing.”

Is the work clearly and accurately presented and does it cite the current literature?

Partly

If applicable, is the statistical analysis and its interpretation appropriate?

Not applicable

Are all the source data underlying the results available to ensure full reproducibility?

Partly

Is the study design appropriate and is the work technically sound?

Yes

Are the conclusions drawn adequately supported by the results?

Yes

Are sufficient details of methods and analysis provided to allow replication by others?

Partly

Reviewer Expertise:

My research area is geotechnical engineering, with a focus on ground improvement techniques

I confirm that I have read this submission and believe that I have an appropriate level of expertise to confirm that it is of an acceptable scientific standard, however I have significant reservations, as outlined above.

# Reviewer report: Reviewer response for version 2

**DOI:** 10.5256/f1000research.208648.r524529

**Published:** 2026-10-13

**Authors:** Abbas J. Al-Taie

**Affiliations:** 1 University of Nahrain, Baghdad, Iraq

This manuscript examines the uplift capacity performance of a structural skirt foundation.

 It tries to improve the uplift capacity by enhancing skirt foundation properties. After the first round of review, the manuscript has been improved. However, the review of the current manuscript indicates that minor revisions are needed before indexing.

 1.         To avoid user confusion, the authors have to ensure the variables/physical symbols shown in Table 2 are fully matched to the nomenclature in the manuscript body text.

 2.         The standard used for the classification of the soil should be shown in Table 1.

 3.         It is necessary to explain whether the ASTM standards were followed to record the structural loading or standard settlement. Otherwise, if other specific conditions are used, they should be stated in the "Methods & Materials."

 4.         The proposed improvement method is for the static uplift condition. The structural durability, the long-term, of the improved properties under cyclic or dynamic loading is critical; including statements about them may strengthen the discussion, or it may be recommended for future work.

Is the work clearly and accurately presented and does it cite the current literature?

Yes

If applicable, is the statistical analysis and its interpretation appropriate?

Not applicable

Are all the source data underlying the results available to ensure full reproducibility?

Yes

Is the study design appropriate and is the work technically sound?

Yes

Are the conclusions drawn adequately supported by the results?

Yes

Are sufficient details of methods and analysis provided to allow replication by others?

Yes

Reviewer Expertise:

Geotechnical Engineering

I confirm that I have read this submission and believe that I have an appropriate level of expertise to confirm that it is of an acceptable scientific standard.

# Reviewer report: Reviewer response for version 1

**DOI:** 10.5256/f1000research.194018.r497522

**Published:** 2026-07-20

**Authors:** Vinay Bhushan Chauhan

**Affiliations:** 1 Madan Mohan Malaviya University of Technology, Gorakhpur, Uttar Pradesh, India

Comments:

  

1. The novelty of the study should be highlighted more clearly. Clearly stating the research gap will strengthen the contribution of the paper.
2. Authors should clarify the number of repetitions performed and discuss the repeatability or variability of the results.
3. The discussion would be stronger if the obtained results were compared quantitatively with findings from previous experimental or numerical studies.
4. The results are presented without any indication of experimental uncertainty or statistical variation.
5. The notation used for geometric parameters should be made consistent. Symbols such as L/B, L/D, and D should be clearly defined and used uniformly throughout the manuscript.
6. The list of symbols appears incomplete. Some parameters and abbreviations used in the manuscript, such as Cu, Cc and LVDT, should also be included.
7. Different terms such as uplift load, uplift force, uplift resistance, and uplift capacity are used throughout the manuscript. It would be better to use one consistent technical term.
8. The fig. Captions could be made more informative by briefly mentioning the important observations or testing conditions.
9. The explanation that the inclined skirt “acts as an anchor” is repeated several times in the Results section. This discussion can be shortened to avoid unnecessary repetition.
10. A careful English language revision is recommended to improve clarity and readability.
11. Conclusions section mainly summarizes experiment results. It would be more valuable if it also discussed the practical engineering implications and the limitations of the present study.

Is the work clearly and accurately presented and does it cite the current literature?

Partly

If applicable, is the statistical analysis and its interpretation appropriate?

Not applicable

Are all the source data underlying the results available to ensure full reproducibility?

Partly

Is the study design appropriate and is the work technically sound?

Partly

Are the conclusions drawn adequately supported by the results?

Partly

Are sufficient details of methods and analysis provided to allow replication by others?

Partly

Reviewer Expertise:

Geotechnical Engineering Rock MEchanics

I confirm that I have read this submission and believe that I have an appropriate level of expertise to confirm that it is of an acceptable scientific standard, however I have significant reservations, as outlined above.

## References

[1] : Adaptive FELA and Machine Learning for Skirted Footing Performance Under Inclined Loading. *Transportation Infrastructure Geotechnology* .2025;12(8) : 10.1007/s40515-025-00753-y 10.1007/s40515-025-00753-y

[2] : Impact of Skirting on the Ultimate Bearing Capacity of Strip Footings on Two-Layered Sandy Soil Slopes.707: 10.1007/978-981-96-7795-5_12157-168 10.1007/978-981-96-7795-5_12

[3] : Seismic bearing capacity of skirted footings on cohesive slopes using limit analysis. *Proceedings of the Indian National Science Academy* .2026; 10.1007/s43538-026-00717-x 10.1007/s43538-026-00717-x

[4] : Bearing Capacity and Failure Mechanisms of Skirted Strip Footings on c–$$phi$$ Slopes Using Limit Analysis. *Transportation Infrastructure Geotechnology* .2025;12(8) : 10.1007/s40515-025-00757-8 10.1007/s40515-025-00757-8

[5] : Finite Element Limit Analysis and Machine Learning Framework for Circular Skirted Mudmat Foundations in Anisotropic Marine Clays. *Transportation Infrastructure Geotechnology* .2026;13(5) : 10.1007/s40515-026-00893-9 10.1007/s40515-026-00893-9

# Response

**Published:** 2026-07-21

**Authors:** Aqeel jafaar

**Affiliations:** civil, University of Baghdad, karbala, Iraq

The authors would like to express their sincere gratitude to the reviewer for their thorough evaluation, valuable comments, and constructive feedback. The reviewer’s insights have been extremely helpful in improving the quality and clarity of our manuscript. Below, we provide point-by-point responses to all the comments and questions raised.

 1- As specialists in geotechnical engineering and skirted foundations, we must be aware of the research gap concerning the response of shallow foundations with skirts to lifting forces. Therefore, the logical sequence of this research will reveal this gap and the feasibility of addressing it. Consequently, we have avoided elaborating on the novelty of the study to prevent distracting the reader from its main focus.

 2-The intensity of the experimental effort and the comprehensive scope of the study are evident through the diversity of parameters studied across 49 models (including variations in L/B dimension ratios and different skirt angles of inclination); the results clearly demonstrate the similarities and differences in the behaviour of shallow skirt foundations, thus eliminating the need to repeat the same experiments.

 3- With full appreciation for your valuable observation, we would like to clarify—as we mentioned previously—that the scarcity of research addressing illuminated shallow foundations subjected to uplift loads made a direct comparison with previous numerical studies impossible at the time of this work (in 2025) due to the lack of similar numerical models at that time covering the same loading conditions and studied parameters. Therefore, the discussion of the results was limited to previous quantitative studies, which simultaneously highlights the importance of this study as a foundational step in this field.

 4- With full appreciation for your valuable feedback, we would like to clarify that the large number of models and experiments conducted in this study (49 models) provided a comprehensive database that enhanced the reliability and empirical certainty of the results. Regarding the statistical analysis, the results were presented and interpreted geometrically and geotechnically through the accompanying tables and graphs. The direct data clearly demonstrates the convergences and divergences, thus achieving the primary objective of the research and enabling the specialist reader to accurately understand the behaviour of the foundations without the need for additional statistical tools.

 5- With full appreciation for your valuable observation, we would like to clarify that retaining some symbols as they appear in previous literature was intentional. This is because some of those studies were conducted on shallow, circular foundations (geometrically dependent on the diameter D), while our current study relies on different geometric parameters (such as length L and width B). Therefore, forcing the standardization of these symbols could lead to confusion or inaccuracy when reviewing and tracing original sources. We would also like to point out that all the symbols you mentioned are known within this manuscript, but you must first download the manuscript file in PDF format to view them.

 6- We thank the reviewer for this valuable suggestion. While all symbols were defined upon their first appearance in the text and tables, we will have update the symbols list to include all relevant symbols.

 7-Given the existence of specific plagiarism standards and percentages imposed by peer-reviewed academic journals, terminological variation and multiple modes of expression emerge as a methodological solution that ensures adherence to those controls without compromising the meaning.

 8- We appreciate the reviewer's valuable suggestion regarding the figure captions. However, as the final layout and structural formatting of the manuscript are governed by the journal's publication guidelines, we have written the captions to provide essential testing conditions and key findings while maintaining full alignment with the journal’s formatting constraints.

 9-We appreciate this valuable observation. We will ensure that this repetition is removed in the revised manuscript. This adjustment will streamline the Results section for better readability without affecting the overall research scope or findings

 10-We appreciate the reviewer’s suggestion. We will conduct a thorough English language revision by a native speaker/professional editing service to enhance the overall clarity, grammar, and readability of the manuscript

 11- Thank you for these highly valuable comments
